# Newly-Obtained Two Organic-Inorganic Hybrid Compounds Based on Potassium Peroxidomolybdate and Dicarboxypyridinic Acid: Structure Determination, Catalytic Properties, and Cytotoxic Effects of Eight Peroxidomolybdates in Colon and Hepatic Cancer Cells

**DOI:** 10.3390/ma15010241

**Published:** 2021-12-29

**Authors:** Adrianna Sławińska, Małgorzata Tyszka-Czochara, Paweł Serda, Marcin Oszajca, Małgorzata Ruggiero-Mikołajczyk, Katarzyna Pamin, Robert Karcz, Wiesław Łasocha

**Affiliations:** 1 Jerzy Haber Institute of Catalysis and Surface Chemistry, Polish Academy of Sciences, Niezapominajek 8, 30-239 Krakow, Poland; adrianna.slawinska@ikifp.edu.pl (A.S.); malgorzata.ruggiero-mikolajczyk@ikifp.edu.pl (M.R.-M.); katarzyna.pamin@ikifp.edu.pl (K.P.); robert.karcz@ikifp.edu.pl (R.K.); 2 Faculty of Pharmacy, Jagiellonian University Medical College, Medyczna 9, 30-688 Krakow, Poland; malgorzata.tyszka-czochara@uj.edu.pl; 3 Faculty of Chemistry, Jagiellonian University, Gronostajowa 2, 30-387 Krakow, Poland; pawel.serda@uj.edu.pl (P.S.); oszajcam@chemia.uj.edu.pl (M.O.)

**Keywords:** peroxidomolybdates, polyoxocompounds, hybrid material, X-ray based crystal structure analysis, thermal decomposition, cyclooctane oxidation, Baeyer–Villiger reaction, cytotoxicity, biochemistry, anti-cancer activity

## Abstract

Two new organic-inorganic hybrid compounds containing dicarboxylic pyridine acids have been obtained and characterized. Both compounds are potassium oxidodiperoxidomolybdates with 2,6-dicarboxylicpyridine acid or 3,5-dicarboxylicpyridine acid moieties, respectively. The chemical formula for the first one is C_14_H_7_K_3_Mo_2_N_2_O_18_ denoted as **K26dcpa**, the second C_7_H_4_K_1_Mo_1_N_1_O_11.5_—**K35dcpa**. Their crystal structures were determined using single crystal (**K26dcpa**) or XRPD—X-ray powder diffraction techniques (**K35dcpa**). The purity of the compounds was confirmed by elemental analysis. Their thermal stability was determined with the use of non-ambient XRPD. In addition, they were examined by IR spectroscopy methods and catalytic activity studies were performed for them. Catalytic tests in the Baeyer–Villiger reaction and biological activity have been performed for eight compounds: **K26dcpa**, **K35dcpa**, and six peroxidomolybdates previously obtained by our group. The anti-proliferative activity of peroxidomolybdenum compounds after 24 h of incubation was studied in vitro against three selected human tumor cell lines (SW620, LoVo, HEP G2) and normal human cells (fibroblasts). The data were expressed as IC_50_ values. The structure of the investigated oxodiperoxomolybdenum compounds was shown to have influence on the biological activity and catalytic properties. It has been shown that the newly-obtained compound, **K35dcpa**, is a very efficient catalyst in the Baeyer–Villiger reaction. The best biological activity results were obtained for *Na-picO* (previously obtained by us), which is a very effective anti-cancer agent towards SW 620 *colorectal adenocarcinoma* cells.

## 1. Introduction

Polyoxometalates (POMs) are a wide group of compounds containing peroxidocompounds as a subgroup. The diverse structures and amazing properties of polyoxometalates are the reason behind the attention devoted to them. The structures of POMs are based on clusters containing transition metal atoms (tungsten, vanadium, niobium, tantalum, or molybdenum) [1,2], surrounded by oxygen atoms. Metal atoms are typically in their highest oxidation state [1]. Single metal center containing clusters of peroxido compounds can be classified as mono-, di-, tri-, and even tetra- peroxido metalates depending on the number of O_2_^2−^ groups. These groups contain relatively weak O-O bonds, and the formal oxidation state of oxygen atoms is −1 [3]. An interesting characteristic of such compounds was presented by Jakob et al., 2007 [4].

POMs possess a wide range of applications described in the literature including catalysis, green chemistry, and chemotherapeutics. Currently, the electrochemical properties of POMs are particularly investigated. For example, Cruz et al. presented new polyoxometalates-based ionic liquids and proved their activity in bleaching and coloration process [5]. Applications of PBA@POM hybrid (PBA: Prussian blue analogue) presented by Wang in 2019, confirmed their excellent electrocatalytic activity [6]. Moreover, there are reports that showed their catalytic properties in olefin epoxidation [7] and oxidation of cyclohydrocarbons [8,9,10,11,12]. An interesting review article regarding the epoxidation of alkanes by molybdenum complexes was presented by Shen in 2019 [13]. The catalytic properties in the Baeyer–Villiger reaction of Keggin-type POMs have also been reported [14]. BV oxidation is an industrially important process for the transformation of cyclohexanone to ε-caprolactone. The latter compound is an important intermediate for manufacturing, among others, biodegradable plastics, pharmaceuticals, herbicides, and other fine chemicals. Although ε-caprolactone is produced on the industrial scale with the use of aggressive and toxic oxygen donor-like peracetic acid, an alternative for the existing processes is a catalytic system in which cyclic ketones can be transformed into lactones, for instance, in the presence of molecular oxygen and aldehyde as a co-catalyst (Mukaiyama conditions) [15]. The proposed method of ε-caprolactone synthesis, due to the in situ peracid formation, eliminates the use of toxic chemicals and limits the formation of hazardous waste. An admirable catalytic activity of oxo-peroxo molybdenum(VI) species on chitosan was shown by Ahmed et al. [16].

Furthermore, the interesting biological activity of POMs is commonly known. Alves published a review report, where he presented antiviral, antibacterial, and antitumor properties of POMs [17]. Moreover, Alves’s review proposed a mechanism behind these activities. Similar antitumor properties of POMs were presented by Saad et al., Yu et al., and Bijec [18,19,20]. The detailed role of amino acid, peptide, and proteins bonding with POMs was described by Arefian et al. in 2017 [21]. In the same year, Paul et al. published a report showing DNA nuclease activity of oxo-peroxomolybdenum(VI) complexes [22]. Saikia et al. used peroxocompounds as inhibitors of calcineurin activity towards RII-phosphopeptide [23]. The wide range of applications of POMs encourages further intensification of synthesis efforts and investigations of new compounds.

The subject showed in this study is a continuation of our previous investigations [10,11,12]. The syntheses procedures are in general the same as in previous cases. The difference lies in the organic substrates, which in this case are dicarboxypyridine acids. The carboxylic acids used in the reaction differ only in the relative position of two carboxylic COO- groups with respect to the N-atoms in the pyridine rings. This factor directly influences the crystal structures of obtained compounds and is also interesting from a crystal engineering point of view. This report highlights the crystal structures and physicochemical properties of the new compounds. Additionally, their catalytic properties are studied in the cyclooctane oxidation reaction as well as in the Baeyer–Villiger reaction (oxidation of cyclohexanone) and were compared against six previously described compounds. All eight listed peroxido-compounds were tested with regard to their biological activity.

## 2. Materials and Methods

### 2.1. Materials

Inorganic chemicals K_2_MoO_4_ and KCl were purchased from POCh (now Avantor Performance Materials, Gliwice, Poland., while 2,6-dicarboxylicpyridine acid, 3,5-dicarboxylicpyridine acid and H_2_O_2_ (30%) were purchased from Sigma-Aldrich, St. Louis, MO, USA.

### 2.2. Syntheses

Compounds were obtained by the reaction of reagents dissolved in H_2_O_2_ (30%, perhydrol) solutions. The first one—**K26dcpa**—was obtained after 2 days as crystals of sufficient quality to be measured using single crystal diffraction methods, while the second one—**K35dcpa**—after 3 days as powder. The course of both chemical reactions was similar to the previously described syntheses [11,12], with the modification of omitting the addition of HCl.

#### 2.2.1. Potassium Oxidodiperoxido(Pyridine-2,6-Carboxylato-N)-Molybdate(VI) (K26dcpa)

A total of 0.02 mol KCl and 0.005 mol K_2_MoO_4_·H_2_O were dissolved in 20 mL of cold H_2_O_2_. Subsequently 0.005 mol of 2,6-dicarboxylicpyridine acid was added to the red solution. All reagents were stirred for one hour. The obtained mixture was filtered and left for crystallization. After 2 days, orange crystals were obtained from the solution. The yield was 63% (1.256 g), C—20.98% (calc. 19.56%), H—1.01% (calc. 1.01%), and N—3.50% (calc. 3.30%).

#### 2.2.2. Dipotassium Bis(µ-Pyridine-N-Oxo-3,5-Carboxylato)Bis(Oxidodiperoxidomolybdate(VI)) Dihydrate (K35dcpa)

A total of 0.02 mol KCl and 0.005 mol K_2_MoO_4_·H_2_O were dissolved in 20 mL of cold H_2_O_2_. Subsequently 0.005 mol of 3,5-dicarboxypyridine acid was added to the red solution. The obtained mixture was filtered and left for crystallization after 3 days, and a large amount of yellow solid was obtained in the solution. The yield was 85% (1.784 g), C—18.95% (calc. 21.06%), H—1.317% (calc. 1.52%), and N—3.21% (calc. 3.51%).

### 2.3. X-ray Powder/Single Crystal Diffraction Data Analysis

The single crystal X-ray diffraction (XRD) studies for **K26dcpa** and the X-ray powder diffraction for **K35dcpa** were carried out in 293 (2) K. The single crystal data was collected using SuperNova diffractometer by Agilent Technologies, currently Rigaku Oxford Diffraction, Frankfurt, Germany, (radiation CuKα, Sapphire 2 detector, graphite monochromator) and the X-ray powder diffraction data using PANalytical X’Pert Pro MPD diffractometer by Malvern Panalytical, Almelo, the Netherlands (radiation CuKα, PIXcel 1-d detector, Bragg-Brentano geometry). The initial analysis of powder diffraction data (phase purity) was performed with High Score software and the PDF-4+ [ICDD, Newtown Square, PA, USA, 2019] database.

The single crystal diffraction-based structure solution and refinement of **K26dcpa** were achieved using SHELXS-97 and SHELXL-2013 programs [24]. The difference Fourier maps were used to determine the location of hydrogen atoms. For all non-hydrogen atoms, refinement of anisotropic displacement parameters was performed. The initial analysis of powder diffraction data (indexing, space group determination) was carried out using the Expo 2014 program [25]. In addition, Expo 2014 managed to locate heavy atoms (Mo and K atoms) using direct methods. An organic molecule of 3,5-dicarboxylicpyridine acid N-oxide was located using the FOX program (direct space methods) [26]. Jana 2006 [27] was used to perform the restrained Rietveld refinement. The final Rietveld refinement plot for **K35dcpa** is presented in Figure S1 in the Supplementary Materials (S). Detailed crystal structure data for **K26dcpa** and **K35dcpa** are presented in Table 1. For the visualization of the crystal structures of the obtained compounds, the Diamond [28] program was used.

### 2.4. IR Measurements

The infrared spectra (IR) investigations were performed using a Bruker VERTEX 70V(Bremen, Germany). The measurements were carried out at room temperature. The analysis of IR spectra was performed with the use of Origin Pro v. 9.1 [29].

### 2.5. X-ray Thermal Decomposition

The thermal stability was examined using Philips X’Pert Pro MPD equipped with a HTK-1200N high-temperature chamber manufactured by Anton-Paar. X-ray data for **K26dcpa** and **K35dcpa** compounds were collected in the 2θ range of 5 to 55° at the following temperatures: 25, 50, 75, 100, 125, 150, 175, 200, 225, 250, 300, 350, 400 °C, and finally at 30 °C. The heating rate was 5 °C/min whereas the cooling rate was 10 °C/min.

### 2.6. Biological Activity

#### 2.6.1. Cell Cultures

Human cell lines were derived from the American Type Cell Culture collection, ATCC (LGC Standards-ATCC (Teddington, UK)), ATCC designations were as follows: BJ, normal adherent human skin fibroblasts, CRL-2522; SW 620, Duke’s type C colorectal adenocarcinoma, CCL-227, LoVo, Duke’s type C grade IV, colorectal adenocarcinoma, CCL-229; and HEP G2, hepatocellular carcinoma, HB-8065. The fibroblasts and HEP G2 cells were grown as monolayer cultures in Eagle’s Minimum Essential Medium, EMEM (Sigma-Aldrich, Seelze, Germany). SW 620 and LoVo cells were kept in Dulbecco’s Modified Eagle’s Medium, DMEM (Sigma-Aldrich, Seelze, Germany). All the media were supplemented with 10% FBS (Eurex, Gdansk, Poland) and with an antibiotic solution (100 IU/mL penicillin, 0.1 mg/mL streptomycin, Gibco Laboratories, Grand Island, NY, USA). The cells were grown in standard cell culture conditions at 37 °C and in a humidified atmosphere of 5% CO_2_ in air.

#### 2.6.2. MTT Assay

MTT, 3-[4,5-dimethylthiazol-2yl]-2,5-diphenyl tetrazolium bromide was purchased from Sigma-Aldrich. For the experiments, suspensions of exponentially dividing cells were placed in 96-well microtiter plates (BD Biosciences, San Jose, CA, USA) at a density of 1 × 10^5^ cells per mL of culture medium. The cells were cultured for 24 h and then the medium in each well was replaced with a new one containing an adequate volume of a stock solution of the tested compounds or an adequate amount of solvent, buffered with PBS (control) (Gibco Laboratories, Grand Island, NY, USA). The cells were exposed to the compounds for 24 h [30]. After incubation, the medium was removed and an MTT assay was performed as described previously [31]. MTT formazan generated during incubation was dissolved and the absorbance was recorded at 550 nm (the reference wavelength was 690 nm) using a microplate reader Infinite M200 Pro, Tecan, Austria. IC_50_ values were calculated from the concentration—response curves (IC_50_ was the concentration [μM/L] of a tested compound required to decrease the cell proliferation to 50% of the control) [32]. All data were expressed as arithmetic mean values with standard deviation (M; ±SD).

### 2.7. Catalytic Activity 

#### 2.7.1. Oxidation of Cyclooctane

The oxidation of cyclooctane was carried out as previously described [9,10,11,12]. The studies were performed in a stainless-steel batch reactor system. Experiments temperature was 120 °C and the applied air pressure was 10 atm. The molar ratio of cyclooctane-to-oxygen was set to 6.5. The oxidation was finished after 6 h by submerging the hot reactor in a cold-water bath. Analyses of reaction products (cyclooctanone and cyclooctanol) were performed by an Agilent 6890 N Gas Chromatograph equipped with an Innowax (30 m) column in the presence of an internal standard (chlorobenzene).

#### 2.7.2. Baeyer–Villiger Reaction

The oxidation of cyclohexanone with molecular oxygen was carried out as previously described [14]. The experiments were performed in a home-made glass reactor equipped with a thermostat. The temperature of the investigations was 40 °C, applied for 5 h under atmospheric pressure. Usually, in a standard experiment, 0.01 mM of catalyst, 4.6 mmol of cyclohexanone, and 14 mmol of benzaldehyde are dissolved in 10 mL of acetonitrile. Due to the reaction conditions (and mixture composition), BV oxidation was performed in heterogeneous conditions. Moreover, the level of oxygen in the reaction mixture was constant and controlled using valves. Analyses of reaction products (percent of consumed cyclohexanone and the percentage yield of ε-caprolactone) were performed by an Agilent 6890 N Gas Chromatograph equipped with an Innowax (30 m) column in the presence of an internal standard (chlorobenzene). Gas chromatography (GC) analyses were carried out using the oven method where the temperature rises from 40 to 170 °C with a step of 20 °C/min. Cyclohexanone, chlorobenzene, and ε-caprolactone were detected after 7.57 min, 12.18 min, and 14.40 min, respectively. The calibration process was carried out for the standard sample before the proper GC analysis. The result of Baeyer–Villiger oxidation is shown as the percentage value defined as the ratio of the obtained amount of the desired product and the theoretical amount of the product resulting from the stoichiometric calculation.

### 2.8. BET

The BET surface area, for peroxidocompounds was measured with nitrogen adsorption at −196 °C on Quantachrome Autosorb-1. Before the experiment, each sample was degassed for 18 h at 25 °C under vacuum to remove adsorbed water and other surface impurities.

## 3. Results

### 3.1. Crystal Structure Data

**Figure 1:**
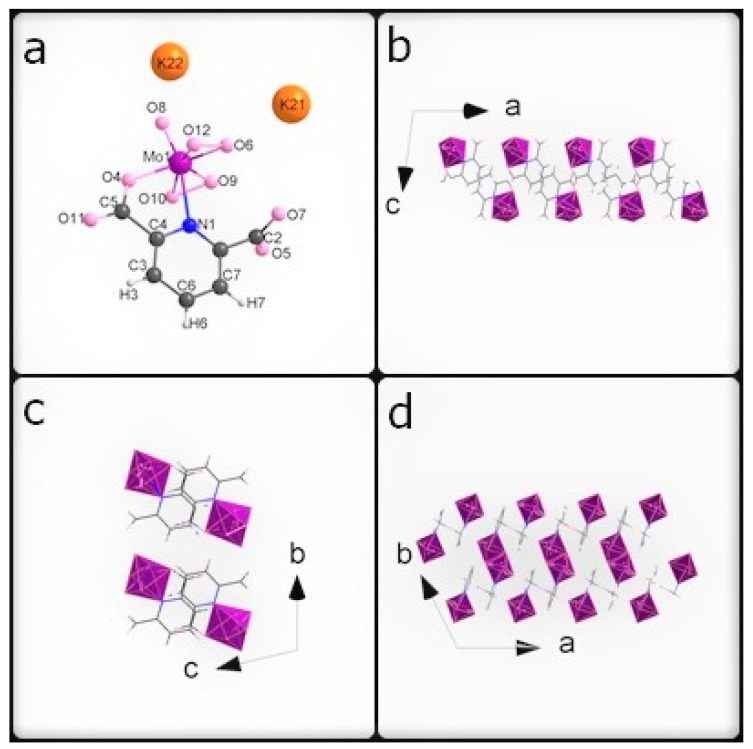
(**a**) The asymmetric unit of **K26dcpa**; packing diagram of **K26dcpa** along (**b**) a axis, (**c**) b axis, and (**d**) c axis.

**Figure 2:**
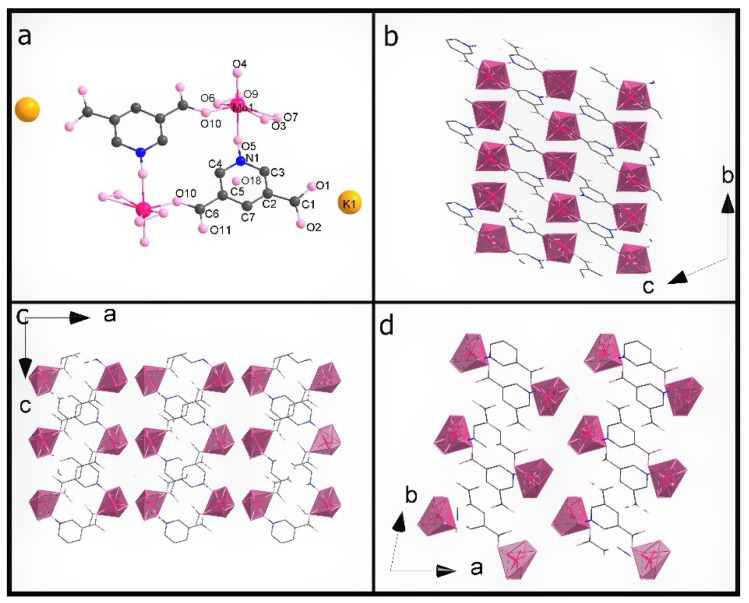
(**a**) The asymmetric unit of **K35dcpa**; packing diagram of **K35dcpa** along (**b**) a axis, (**c**) b axis, and (**d**) c axis.

The most important crystal data are presented in Table 1. Table S1 shows a list of selected bond lengths. Figure 1a–d shows the **K26dcpa** structure while the structure of **K35dcpa** is presented in Figure 2a–d.

Both compounds are inorganic-organic hybrids and both are potassium salts too. The pentagonal bipyramids around the metal centers each contain two peroxo groups and one apical terminal oxygen in both cases.

The first compound is similar to the compound obtained by Mares in 1978 [33]. In **K26dcpa**, the anion contains an inorganic part (pentagonal bypiramid) connected with the organic ligand (2,6-dicarboxypyridine acid). The linking atoms are N1 belonging to the aromatic ring with N1-Mo distance 2.4054 (17) Å, and O4 from the carboxylic group with the O4-Mo distance of 2.0332 (20) Å. The pentagonal bipyramid is formed by the molybdenum atom surrounded by six oxygen atoms and one nitrogen atom. The distance between the apical terminal oxygen atom and molybdenum atom position is 1.6898 (17) Å. The Mo atom is shifted from the equatorial plane of the pentagonal bipyramid by 0.3172 (2) Å. The angle between the equatorial plane and aromatic ring plane is 85.445(73)°.

The second compound, **K35dcpa**, contains dimeric anions. Connections between the inorganic and organic parts are formed by two oxygen atoms. One of the linking atoms belongs to one of the carboxylic groups present in the structure (O10-Mo distance is 1.99 (6) Å), and the second linking atom belongs to the N-oxide-group with a O5-Mo distance of 2.20 (9) Å. Pentagonal bipyramids are formed around the molybdenum atoms by seven oxygen atoms (MoO_7_). The distance between the molybdenum atom and apical terminal oxygen atom (Mo=O4) is 1.68 (10) Å. In addition, the molybdenum atom is moved from the equatorial plane of the pentagonal bipyramid by 0.46 (2) Å. The equatorial plane and aromatic ring plane are twisted relative to each other by 46 (2)°. Moreover, the corners of the pentagonal bipyramids create channels along the b axis.

Interestingly, **K35dcpa** behaves similarly to 3-cpa, a structure containing nicotinic acid, previously observed and described by us [10]. 3,5-dcpa forms a centrosymmetric cyclic anion (see Figure 2d) with the 3,5-dcpa acid molecule oxidized to an N-oxide in a manner analogous to the nicotinic acid molecule in 3-cpa.

A closer inspection of the organic parts of the structures reveals π-π interactions for both 2,6-dicarboxylicpyridine (1) acid or 3,5-dicarboxylicpyridine (2) acid rings. The shortest distance between atoms of neighboring aromatic rings is 3.3079 (50) Å for **K26dcpa** and 3.61 (10) Å for **K35dcpa**. The distances between planes based on adjacent aromatic rings are 3.40 (6) Å and 3.58 (3) Å, respectively.

#### Peroxidocompounds Selected for Biological and Catalytic Investigations

Table 2 presents all peroxidocompounds tested for biological and catalytic activity (Baeyer–Villiger reaction). Descriptions of six of the studied structures have been previously published by our group [10,11,12]. The inorganic parts of each compound contain the molybdenum atom surrounded by seven oxygen atoms (except for **K26dcpa**, where one of the oxygen atoms is replaced with a nitrogen atom).

### 3.2. IR Spectra

**Figure 3:**
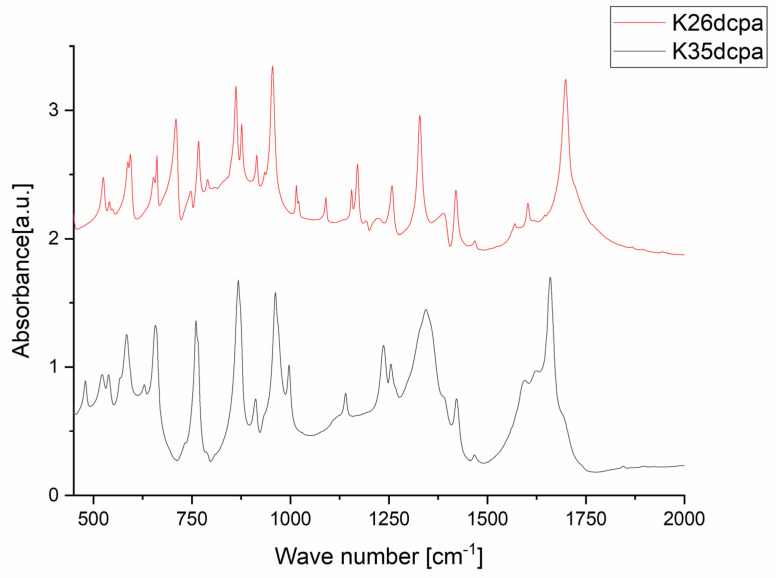
IR spectra of the compounds **K26dcpa** and **K35dcpa**. N-oxide and Mo-N vibrations listed in Table 3.

The purpose of the IR studies was to identify and confirm the presence of oxo- and peroxo-bonds, N-oxide vibrations, and Mo-N vibrations. The wavenumbers assigned to specific vibration types for both studied compounds are listed in Table 3, and the registered spectra are shown in Figure 3. The spectra of both compounds contain similar bands, which correspond to the vibrations of a similar inorganic part (ν(Mo=O); ν_sym_(O-O); ν_sym_(Mo-(O)_2_); ν_asym_(Mo-(O)_2_)). Moreover, identification of the N-oxide vibration and Mo-N vibration can be instrumental in the verification of the synthesis product and the correctness of the XRPD structure determination. Other bands, not included in Table 3, can be assigned to the organic parts of each compound [3,9,10,11,12,34,35,36,37].

### 3.3. Thermal Decomposition

**Figure 4:**
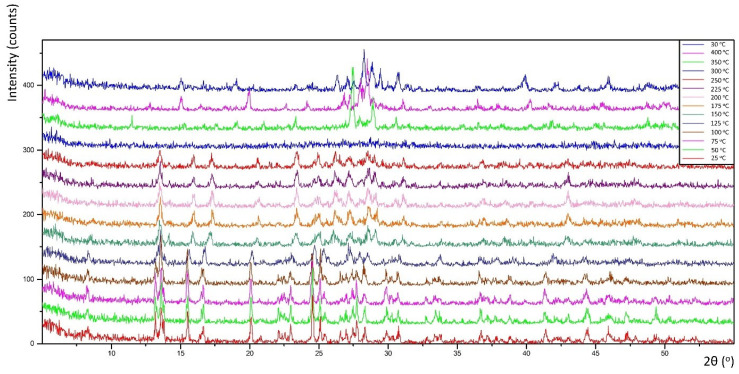
Thermal decomposition for the compound **K26dcpa** (see text for description).

**Figure 5:**
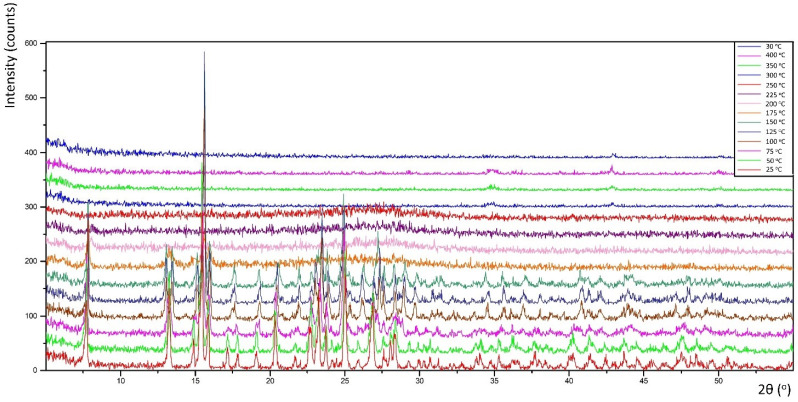
Thermal decomposition for the compound **K35dcpa** (see text for description).

Thermal decomposition investigations were carried out to reveal the relation between thermal stability and molecular structure. Obtained diffractograms (non-ambient XRPD) verified the stability of the **K26dcpa** compounds up to 125 °C and **K35dcpa** compounds up to 75 °C. For **K26dcpa,** gradual decomposition was observed in the 150–250 °C range. The amorphous phase was observed at 300 °C. Subsequently, potassium molybdate phases were detected [pdf-4+ 00-023-0489]; [pdf-4+ 00-021-0663]. These phases (K_2_MoO_4_ and K_2_Mo_2_O_7_) were stable after cooling the studied sample to 30 °C (Figure 4). In the case of **K35dcpa**, gradual degradation was observed in the 100–150 °C range. Above these temperatures, the compound decomposed to an amorphous phase (Figure 5).

### 3.4. BET: Specific Area and Porosity Determination

The aim of the BET measurements was to determine the specific surface area (S_SA_), pore sizes, and volume. All results are presented in Table 4. All of the compounds show very low S_SA_, which results from their crystal structures. In three cases, the determination of specific surface area was not possible; the S_SA_ was under 1 m^2^/g for **K35dcpa**, *K-nicO*, and *NH_4_-picO*. These values are not typical for potential catalysts for heterogenous catalysis, but are not uncommon for such compound types (heteropolyacids) [38]. Moreover, six of the eight samples showed various pore sizes. The pore volume (BJHdes method) ranged from 0.0093 cm^3^/g to 0.060 cm^3^/g and the specific surface area (related to pore volume) ranged from 0.71 m^2^/g to 3.3 m^2^/g.

## 4. Biological Activity

### Anti-Proliferative Activity of Compounds against Normal and Tumor Cells

An in vitro experimental model was used to determine whether the newly synthesized compounds exert biological activity in human cells. Four different human cell lines with normal characteristics (fibroblasts) and also with cancer origin (*hepatocellular carcinoma*, HepG2, and *colorectal adenocarcinomas* LoVo and SW 620) were employed. MTT colorimetric assay was used to assess the anti-proliferative activity of tested compounds after 24 h of incubation and the collected data were expressed as IC_50_ values [µM/L].

No oxodiperoxidocompounds were previously tested for biological activity. As shown in Table 5, all synthesized compounds had anti-proliferative activity in cultured human cells. In general, the compounds showed enhanced inhibitory potency towards colon cancer cell lines (SW 620 and LoVo) compared with normal cells, while the effect on hepatic tumor cells was minor. This may suggest tissue-specificity of the compounds, but a more extensive study is necessary to confirm such trends. It might be speculated that the biological activity depends on the organic part of each compound because they determine the crystal structures of the compounds. However, five out of eight compounds differ only in the organic part and there is no statistical difference in the biological activities between these complexes. On the other hand, the data reveals the significant impact of the type of the cation on the biological activity. Three (*K-picO*, *Na-picO*, and *NH_4_-picO*) out of eight compounds differ in the type of the cation (K^+^, Na^+^, NH_4_^+^), while the organic part (N-oxide-picolinic acid) remains the same. It should be noted that the *Na-pico* complex exerted the highest inhibitory effect, with an IC_50_ value of 26.77 ± 8.5 μM/L (Table 5). At the same time, *Na-pico* was much less cytotoxic towards normal cells (IC_50_ value for fibroblasts was 143.35 ± 18.6 μM/L). Therefore, we may speculate that the kind of cation which stabilizes the structure is an important factor influencing the interaction of the *Na-picO* compound with living cells. The cellular context of this finding is of interest and the specific mechanism behind it will be further elucidated.

## 5. Catalytic Activity

### 5.1. Oxidation of Cyclooctane

**Figure 6:**
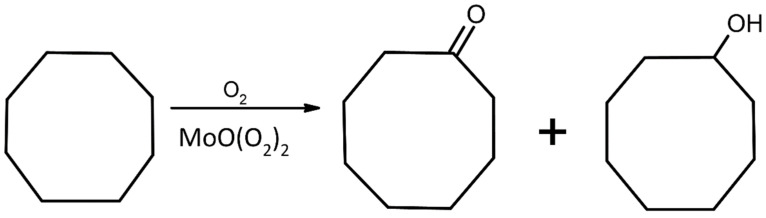
Scheme of cyclooctane oxidation with molecular oxygen and peroxidomolybdates as catalysts.

The newly obtained compounds were used as catalysts cyclooctane oxidation with molecular oxygen. Figure 6 shows a general scheme of this reaction.

Table 6 contains the results of the cyclooctane oxidation reactions that were tested for **K26dcpa** and **K35dcpa**. Six other similar peroxidomolybdenum compounds that have been tested and previously published are listed here as well for comparative purposes. Two new compounds show very low catalytic activity towards this reaction. We may suppose that these compounds are not stable at the temperature of the reaction (120 °C) based on the nonambient-XRPD results.

### 5.2. Baeyer-Villiger Reaction

**Figure 7:**
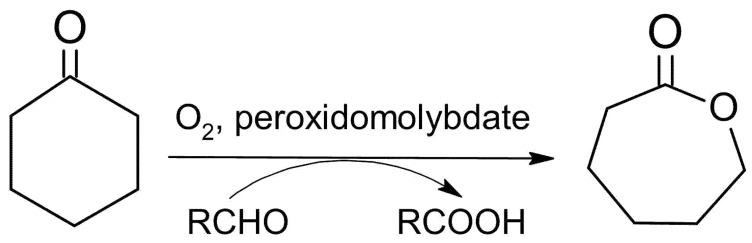
Scheme of catalytic Baeyer–Villiger (BV) cyclohexanone oxidation with molecular oxygen and peroxidomolybdates as catalysts.

**Figure 8:**
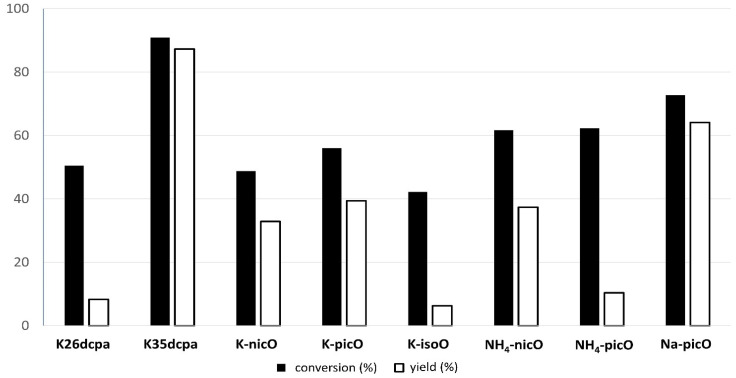
The BV oxidation of cyclohexanone with peroxidocompounds as catalysts in the first run.

The obtained compounds were used as catalysts in the Baeyer–Villiger oxidation of cyclohexanone with molecular oxygen to ε-calprolactone. Figure 7 shows a general scheme of the Baeyer–Villiger reaction. Table 7 presents the results of catalytic activity studies, and Figure 8 presents the results of the first run. Figures S1–S5 present the comparison of catalytic activity in runs I, II, and III for each individual sample.

In the catalytic reaction of cyclohexanone oxidation, eight peroxidomolybdenum compounds were tested: Five compounds with potassium cation, two with ammonium cation, and one with sodium cation. The investigated compounds are presented in Table 2. Seven out of the eight compounds showed a high achieved conversion of 45% and more, while, on the other hand, the yield of the reaction was varying. Based on the selectivity defined as the yield to conversion ratio, we decided to test five promising compounds where the selectivity reached above 50% in more catalytic cycles. All data are presented in Table 7, whereas the graphs presenting three runs for each of the five compounds are shown in the Supplementary Materials (Figures S4–S8). In addition, the TON value was calculated for all data (see Table 7).

The highest conversion degree was observed for **K35dcpa** sample (90.9%), which was characterized by the highest yield of the reaction (89.7%) and the highest TON value (412.62) in the first run. In addition, for this compound the results of the second run were equally high. It is interesting that compounds with N-oxide bonds (N → O-Mo) have higher catalytic activities than compounds with N-Mo bonds (**K35dcpa** is more active than **K26dcpa**). *Na-picO* was second: Conversion (73.5%), yield (62.3%), and TON (286.58). This compound was the best in the group containing monocarboxylicpyridine acid. Detailed information about runs I, II, and III for selected compounds are presented in the Supplementary Materials, Section S4. Catalytic parameters for subsequent reaction runs are quite stable, which makes them promising objects for further tests.

Moreover, it was also proven that in the presence of K35dcpa but without molecular oxygen, the reaction does not proceed. The resulting acyl radical, formed after the abstraction of hydrogen from the aldehyde molecule by Mo6+, reacts with molecular oxygen forming acylperoxy radical [14]. The latter abstracts hydrogen from the following aldehyde molecule to afford an in situ formation of peracid and acyl radical. Peracid is a key molecule to produce the Criegee complex, which subsequently decomposes with the formation of ε-caprolactone.

In addition, Sections S4 and S5 show the comparison of XRPD patterns, IR spectra, and the chemical analysis results collected before and after the BV reaction. These investigations aimed to determine the stability of catalysts in the conditions of the BV reaction.

Structure changes for *NH_4_-nicO*, **K35dcpa, K26dcpa**, and *Na-picO* were not observed, while some changes occurred in the case of *K-nicO* and *K-picO,* although chemical analysis and IR spectra indicate only small changes in ‘structure skeletons’. Probably, the BV reaction mostly caused dehydration of the catalyst, similar to that investigated in this study, see Supplementary Materials for details.

Moreover, in Section S3, the TG/DSC investigation for **K35dcpa** and **K26dcpa** is shown. The tests show rapid decomposition (in the form of an explosion) of the **K35dcpa** compound at 160 °C. We recommend caution in testing similar compounds. 

In conclusion, the tested materials are stable under the BV reaction conditions and are also promising catalysts. However, the specific area and ‘pores size’ (see Section 3.4) are similar for all compounds. There is no definite difference between compounds of different cations (K, Na, and NH_4_). There are no definite differences between *K-nicO* and *K-picO.*

The best catalyst is **K35dcpa** with two carboxyl groups.

The weakest catalysts are:

- *K-isoO*, polymeric structure,
- **K26dcpa**, without N → O-Mo bonds.

The polymeric structure of the compound *K-isoO* as well as the presence of substituents in the 2,6- positions in K26dcpa may block access to the molybdenum centers. The multiple non-blocking -COO groups may support the peroxycarboxylic groups formation and increase catalytic activity. However, the collected experimental material (8 compounds) do not seem to be sufficient for a broader, credible generalization.

## 6. Discussion & Conclusions

Two oxidodiperoxidomolybdenum compounds containing dicarboxypyridine acid were obtained and characterized. Using 2,6-dicarboxypyridine acid we can obtain a simple, monomeric cluster, while using 3,5-dicarboxylicpyridine acid cyclic, dimeric clusters can be formed. In addition, using 3,5-dicarboxypyridine acid, we obtained the N-oxide form of this acid in the structure. In the case of compounds with 2,6-dicarboxypyridine, we observed a direct bond of the molybdenum atom to nitrogen. This is due to the different relative position of the COO^−^ group with respect to the N atom in the aromatic ring. Both compounds were stable up to relatively low temperatures of 125 °C and 75 °C, respectively.

Eight peroxidomolybdenum compounds were tested to compare their catalytic properties and biological activity. Five of them differ only in the organic part of the compounds. The other three compounds also differ in the cation stabilizing the structure.

The two newly-obtained compounds **K26dcpa** and **K35dcpa** were used as a catalyst in the reaction of cyclooctane oxidation. Both compounds showed very low catalytic activity. The reason for this observation is the high temperature of the reaction (120 °C), which is problematic because both peroxidomolybdates are not stable at this temperature.

The obtained compounds were used as catalysts in the Baeyer–Villiger oxidation of cyclohexanone with molecular oxygen to ε-calprolactone. Among the investigated compounds, potassium oxodiperoxomolybdates are the most promising group of compounds. Moreover, the organic part also affects catalytic properties. The best results were obtained for **K35dcpa** (dipotassium bis(µ-pyridine-N-oxo-3,5-carboxylato)bis(oxidodiperoxidomolybdate(VI)) dihydrate) and *Na-picO* (sodium oxidodiperoxido(pyridine-N-oxo-2-carboxylato)molybdate(VI) dihydrate). Due to their stability, both of these compounds appear to be promising candidates for practical testing in the BV reaction. In addition, the number of carboxylic groups has an influence on the catalytic properties: A compound with two carboxylic groups is nearly twice more active than its counterpart with one carboxylic group and the same type of cation.

All oxidoperoxidomolybdenum compounds were active against tumor cells and showed selectivity towards colon cancer cells. The best results were obtained for *Na-picO* (Sodium oxidodiperoxido(pyridine-N-oxo-2-carboxylato)molybdate(VI) dihydrate), which was exceptionally active against colon cancer cells with aggressive characteristics. At the same time, this compound was not toxic towards normal cells which makes it an interesting candidate for further modifications and investigation of specific anti-cancer properties. To summarize, the biological activity of the described compounds depending on the organic moiety decreased in the following sequence:

- Compounds with picolinic part or a 2,6-dicarboxypiridinic part (the most active),
- Compounds containing nicotinic or a 3,5-dicarboxypiridinic part,
- Compounds with an isonicotinic part (the least active).

Based on the described investigations, it is claimed that the most important factor relating to the peroxidomolybdenum inorganic-organic structure, as well as catalytic or biological activity is the complicated synergy between the oxidodiperoxido group, organic moiety, and cation.

## Figures and Tables

**Table 1:** Summary of crystal data of the investigated compounds.

Compound Code, (XRD Technique)	K26dcpa, Single Crystal	K35dcpa, Powder
**Chemical formula**	C_14_H_7_K_3_Mo_2_N_2_O_18_	* C_7_H_3_K_1_MoN_1_O_11.5_
**Structural formula,**	K_3_[MoO(O_2_)_2_C_5_H_3_N(COO)(COOH_0.5_)]_2_	K[MoO(O_2_)_2_C_5_H_3_NO(COO)_2_] 1/2H_2_O
**MW (g/mol)**	800.387	420.1
**T(K)**	293 (2)	293 (2)
**Wavelength, [Å]**	CuKα: 1.54187	CuKα: 1.54187
**Crystal system, SG**	triclinic, P −1	triclinic, P −1
**Cell parameters:**		
**a[Å]**	7.2171(3)	11.7543(15)
**b[Å]**	8.0297(4)	7.9665(10)
**c[Å]**	10.9099(3)	7.3364(9)
**α[°]**	101.131(3)	113.954(11)
**β[°]**	91.968(3)	94.799(12)
**γ[°]**	115.509(4)	77.705(12)
	**K26dcpa**	**K35dcpa**
**V(Å^3^)**	554.93(4)	613.41(15)
**Z, calculated density (g/cm^3^)**	1, 2.3949	2, 2.2747
**Absorption coefficient (mm^−1^)**	15.314	12.448
**F(000)**	391	410
**Theta range**	4.172–77.447	5.007–79.992
**Limiting indices**	−8 ≤ h ≤ 8; −10 ≤ k ≤ 10; −13 ≤ l ≤ 13	−9 ≤ h ≤ 8; −6 ≤ k ≤ 6; 0 ≤ l ≤ 6
**Reflections collected/unique**	20,106/2270	5712
**Completeness to theta**	77.447, 96.8%	100% (powder sample)
**Absorption correction**	None	Capillary, calc. for cylindrical sample
**Refinement method**	F^2^ (Fsqd)	Rietveld
**Data/restraints/parameters**	2270/0/173	5712/38/91
**Goodness of fit on F2**	1.088	4.85
**Final R indices [I > 2σ(I)]**	R1 = 4.75, wR2 = 13.25	-
**R indices (all data)**	R1 = 4.79, wR2 = 13.32	Rp = 0.0832, Rwp = 0.1002
**Extinction coefficient**	None	-
**Largest difference peak and hole (eA^−3^)**	2.054; −2.378	−0.94; 1.18
**CCDC**	2119275	2118837

*—due to XRPD limitations, five H atoms in the unit cell (from 3/2 water molecules and -COOH groups) were not located.

**Table 2:** Structural data of the compounds tested for biological and catalytic activity (cyclooctane oxidation, and Baeyer–Villiger reaction).

Comp. Name, Cation	Organic Part of the Structure, Anion	Type of Anion	MW (g/mol)	Date of Publication, CCDC Number Ref.
*K-nicO*, K	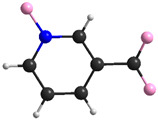	Dimeric, dinuclear cluster	371.15	2017, 1473954 [11]
*K-picO*, K	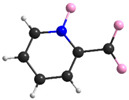	Monomeric cluster	353.14	2017, 1473958 [11]
*K-isoO*, K	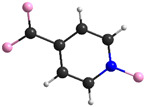	Polymeric anion	369.1	2020, 1943663 [12]
**K-26dcpa, K**	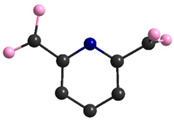	Monomeric cluster	400.2	New, 2119275
**K-35dcpa, K**	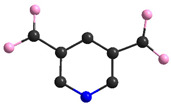	Dimeric, dinuclear cluster	420.1	New, 2118837
*NH_4_-nicO*, NH_4_	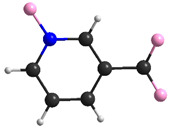	Dimeric, dinuclear cluster	664.1562	2013, 848660 [10]
*NH_4_-picO,* NH_4_	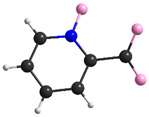	Monomeric cluster	332.08	2017, 1473955 [11]
*Na-picO*, Na	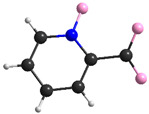	Monomeric cluster	373.04	2017, 1473959 [11]

**Table 3:** IR spectra vibrations and band assignments of investigated oxodiperoxidomolybdates (based on literature data: [3,9,10,11,12,34,35,36,37]). Vs-very strong, s-strong, m-medium, and w-weak.

Compound	ν(Mo=O)	ν_sym_(O-O)	ν_sym_(Mo-(O)_2_)	ν_asym_(Mo-(O)_2_)	(N-Oxide) Vibrations	(Mo-N) Vibrations
**K26dcpa**	955 vs	877 s, 862 vs	594 m, 588 m	541 w	-	1015 w
**K35dcpa**	962 vs	868 vs	585 s	539 m	480 w	-

**Table 4:** N_2_ physisorption-derived parameters characterizing the obtained samples (S_SA_—specific surface area).

Compound	S_SA_ (m^2^/g)	Pores Size BJH_des_ (Å)	Pores Volume BJH_des_ (cm^3^/g)
**K26dcpa**	0.71	41, 74	0.012
**K35dcpa**	without measurement *	29, 37, 53, 88	0.016
*K-nicO*	without measurement *	53, 89, 276	0.011
*K-picO*	1.2	62	0.028
*K-isoO*	1.4	90, 41	0.041
*NH_4_-nicO*	3.3	47, 74, 179	0.060
*NH_4_-picO*	without measurement *	37, 73, 112, 281	0.0093
*Na-picO*	2.8	62	0.043

* S_SA_ below 1 m^2^/g.

**Table 5:** IC_50_ values [μM/L] of synthesized compounds at inhibiting the proliferation of normal human fibroblasts (BJ) and human tumor cells (Hep G2, SW 620, LoVo) as determined by the MTT assay. Results are means ± SD (*n* = 3).

	Normal Cells	Human Tumor Cells		
		*Hepatocellular Carcinoma*	*Colorectal Adenocarcinomas*	
	Fibroblast	Hep G2	LoVo	SW 620
*K-nicO*	138.75 ± 2.6	131.17 ± 12.4	79.85 ± 3.3	66.2 ± 2.1
*K-picO*	135.8 ± 21.6	157.2 ± 25.7	89.91 ± 4	66.2 ± 6.5
*K-isoO*	139.75 ± 27.6	129.13 ± 9.2	86.79 ± 3.6	68.07 ± 18.6
**K-26dcpa**	149.65 ± 16.2	139.75 ± 3.6	62.98 ± 2.8	55.62 ± 7
**K-35dcpa**	148.3 ± 15.7	168 ± 26.7	97.51 ± 3	83.07 ± 1.9
*NH_4_-nicO*	145.7 ± 17.5	153.83 ± 15.7	86.64 ± 5.2	67.47 ± 13.7
*NH_4_*-*picO*	132.15 ± 13.6	122.17 ± 15.2	65.8 ± 16	62.11 ± 7.1
*Na*-*picO*	143.35 ± 18.6	132.25 ± 7.6	78.66 ± 4.4	26.77 ± 8.5

**Table 6:** Oxidation of cyclooctane using molybdenum complexes.

Catalyst Number and Code	Cyclooctanone (%)	Cyclooctanol (%)	Cyclooctanone + Cyclooctanol	Cyclooctanone/Cyclooctanol
**K26dcpa**	0.74	0.64	1.38	1.16
**K35dcpa**	0.97	0.64	1.61	1.52
Examples from our previous studies [9,10,11,12]				
*K-nicO*	5.1	4.1	9.2	1.2
*K-picO*	13.6	13.6	27.2	1.0
*K-isoO*	1.0	0.8	1.8	1.25
*NH_4_-nicO*	32.9	20.2	53.1	1.6
*NH_4_-picO*	32.0	18.4	50.4	1.7
*Na-picO*	12.0	12.8	24.8	0.9

**Table 7:** Result of oxidation of cyclohexanone with molecular oxygen to ε-calprolactone.

Catalyst Symbol	Run No.	Conversion (%)	Yield (%)	Selectivity (%)	TON ^a^
**K26dcpa**	I	50.5	8.3	16.44	38.18
	I	90.9	87.3	98.68	401.58
**K35dcpa**	II	91.8	90.7	98.80	417.22
	III	71.2	59.2	83.15	272.32
	I	48.8	32.9	67.42	151.34
*K-nicO*	II	38.1	24.5	64.30	112.7
	III	49.4	24.0	48.58	110.4
	I	56.00	39.40	70.36	181.24
*K-picO*	II	49.20	18.80	38.21	86.48
	III	55.90	11.60	20.75	53.36
*K-isoO*	I	42.20	6.30	14.93	28.98
	I	61.70	37.40	60.62	172.04
*NH_4_-nicO*	II	42.40	29.60	69.81	136.16
	III	45.70	13.20	28.88	60.72
*NH_4_-picO*	I	62.30	10.40	16.69	47.84
	I	72.7	64.1	84.76	294.86
*Na-picO*	II	70.4	68.3	97.02	314.18
	III	56.7	51.9	91.53	238.74

^a^ moles of reactant converted per mole of catalyst.

## Data Availability

Crystal structure data have been deposited at the Cambridge Crystallographic Data Centre and allocated the deposition numbers CCDC 2119275 (**K26dcpa**), CCDC 2,118,837 (**K35dcpa**). These data can be obtained free of charge via http://www.ccdc.cam.ac.uk/conts/retrieving.html, (accessed on 1 December 2021), or from the Cambridge Crystallographic Data Centre, 12 Union Road, Cambridge CB2 1EZ, UK; fax: (+44)-1223-336-033; or e-mail: deposit@ccdc.cam.ac.uk. Supplementary data associated with this article can be found, in the online version.

## Supplementary Materials

The following supporting information can be downloaded at: https://www.mdpi.com/article/10.3390/ma15010241/s1, Figure S1: **K35dcpa**—Final Rietveld refinement plots (JANA2006 program). Gray bar indicates the so-called ‘excluded region’—excluded due to the presence of diffraction lines from the ‘capillary system’; Figure S2: TG/DSC investigation for **K26dcpa**; Figure S3: TG/DSC investigation for **K35dcpa**; Figure S4: The BV oxidation of cyclohexanone with **K35dcpa** as catalyst in I, II, III run; Figure S5: The BV oxidation of cyclohexanone with *K-nicO* as catalyst in I, II, III run; Figure S6: The BV oxidation of cyclohexanone with *K-picO* as catalyst in I, II, III run; Figure S7: The BV oxidation of cyclohexanone with *NH4-nicO* as catalyst in I, II, III run; Figure S8: The BV oxidation of cyclohexanone with *Na-picO* as catalyst in I, II, III run; Figure S9: PXRD before and after BV reaction for *NH_4_-nicO*; Figure S10: PXRD before and after BV reaction for *K-nicO*; Figure S11: PXRD before and after BV reaction for *K-picO*; Figure S12: PXRD before and after BV reaction for **K35dcpa**; Figure S13: PXRD before and after BV reaction for *Na-picO*; Figure S14: PXRD before and after BV reaction for **K26dcpa**; Figure S15: IR spectra of the compounds *K-nicO* before and after BV reaction; Figure S16: IR spectra of the compounds *K-picO* before and after BV reaction; Table S1: Selected bond lengths (Å) in the studied compounds; Table S2: Result of elemental analysis for *K-nicO* and *K-picO* before and after BV reaction.
