# Supplementary material for: Newly-Obtained Two Organic-Inorganic Hybrid Compounds Based on Potassium Peroxidomolybdate and Dicarboxypyridinic Acid: Structure Determination, Catalytic Properties, and Cytotoxic Effects of Eight Peroxidomolybdates in Colon and Hepatic Cancer Cells
Source: Materials (Basel). 2021 Dec 29;15(1):241. doi: 10.3390/ma15010241 (PMC8746204; doi:10.3390/ma15010241)
Supplement: Supplementary file 1 [file materials-15-00241-s001.zip › materials-1480475-supplementary.pdf]

<u>**Supplementary Materials**</u>

Newly-Obtained Two Organic-Inorganic Hybrid Compounds Based on Potassium Peroxidomolybdate and Dicarboxypyridinic Acid: Structure Determination, Catalytic Properties, and Cytotoxic Effects of Eight Peroxidomolybdates in Colon and Hepatic Cancer Cells

Adrianna Sławińska [1], Małgorzata Tyszka-Czochara [2], Paweł Serda [3], Marcin Oszajca [3], Małgorzata Ruggiero-Mikołajczyk [1], Katarzyna Pamin [1], Robert Karcz [1] and Wiesław Łasocha [1,3,*]

[1] Jerzy Haber Institute of Catalysis and Surface Chemistry, Polish Academy of Sciences, Niezapominajek 8, 30-239 Krakow, Poland; adrianna.slawinska@ikifp.edu.pl (A.S.); malgorzata.ruggiero-mikolajczyk@ikifp.edu.pl (M.R.-M.); katarzyna.pamin@ikifp.edu.pl (K.P.); robert.karcz@ikifp.edu.pl (R.K.)
[2] Faculty of Pharmacy, Jagiellonian University Medical College, Medyczna 9, 30-688 Krakow, Poland; malgorzata.tyszka-czochara@uj.edu.pl
[3] Faculty of Chemistry, Jagiellonian University, Gronostajowa 2, 30-387 Krakow, Poland; pawel.serda@uj.edu.pl (P.S.); oszajcam@chemia.uj.edu.pl (M.O.)
* Correspondence: lasocha@chemia.uj.edu.pl

Register
1. Selected lengths of bonds for **K26dcpa** and **K35dcpa**.
2. Rietveld refinement plots for **K35dcpa**.
3. TG/DSC investigations for **K35dcpa** and **K26dcpa**
4. Catalysis for selected compounds comparing I, II, III run.
5. X-ray diffraction, IR spectroscopy, an elemental analysis for selected compounds *K-nicO*, *K-picO*, *NH_4-nicO*, **K35dcpa**, **K26dcpa** and *Na-picO* before and after BV reaction.

1. Selected lengths of bonds for K26dcpa and K35dcpa.

Table S1. Selected bond lengths (Å) in the studied compounds.

**K26dcpa**		**K35dcpa**	
Mo1 – O8	1.6898(17)	Mo1-O4	1.68(11)
Mo1 -O6	1.9176(24)	Mo1-O6	1.92(9)
Mo1 -O9	1.9239(25)	Mo1-O3	1.93(6)
Mo1-O12	1.9484(27)	Mo1-O5	2.20(9)
Mo1-O10	1.9813(28	Mo1-O9	2.02(8)
Mo1-O4	2.0332(20)	Mo1-O10	1.99(6)
Mo1N1	2.4054(17)	Mo1-O3	1.93(6)
O4-O11	2.2286(27)	O6-O9	1.36(8)
O5-O7	2.2385(39)	O7-O3	1.34(11)
O6-O12	1.4728(32)	O18-O7	2.24(11)
C2-O5	1.2314(46)	C1-O1	1.28(7)
C2-O7	1.2806(43)	C1-O2	1.27(7)
C5-O11	1.2105(39)	O5-N1	1.33(8)
C5-O4	1.3101(30)		

2. Rietveld refinement plots for K35dcpa.

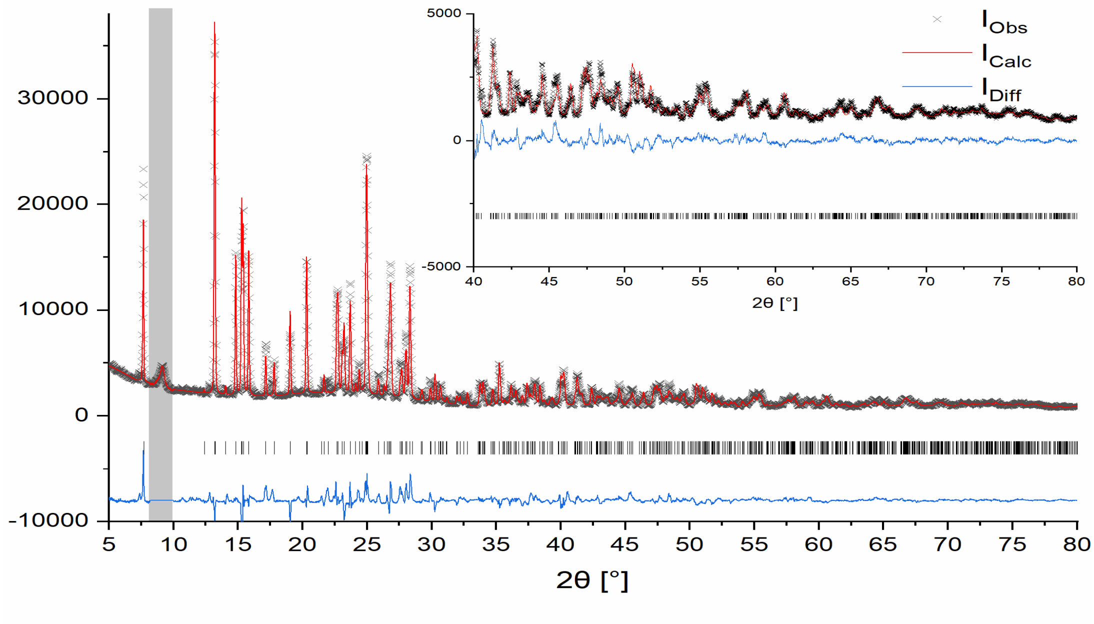

Figure S1. K35dcpa - Final Rietveld refinement plots [JANA2006 program]. Gray bar indicates the so-called 'excluded region' - excluded due to the presence of diffraction lines from the 'capillary system'.

3. TG/DSC investigations for K35dcpa and K26dcpa.

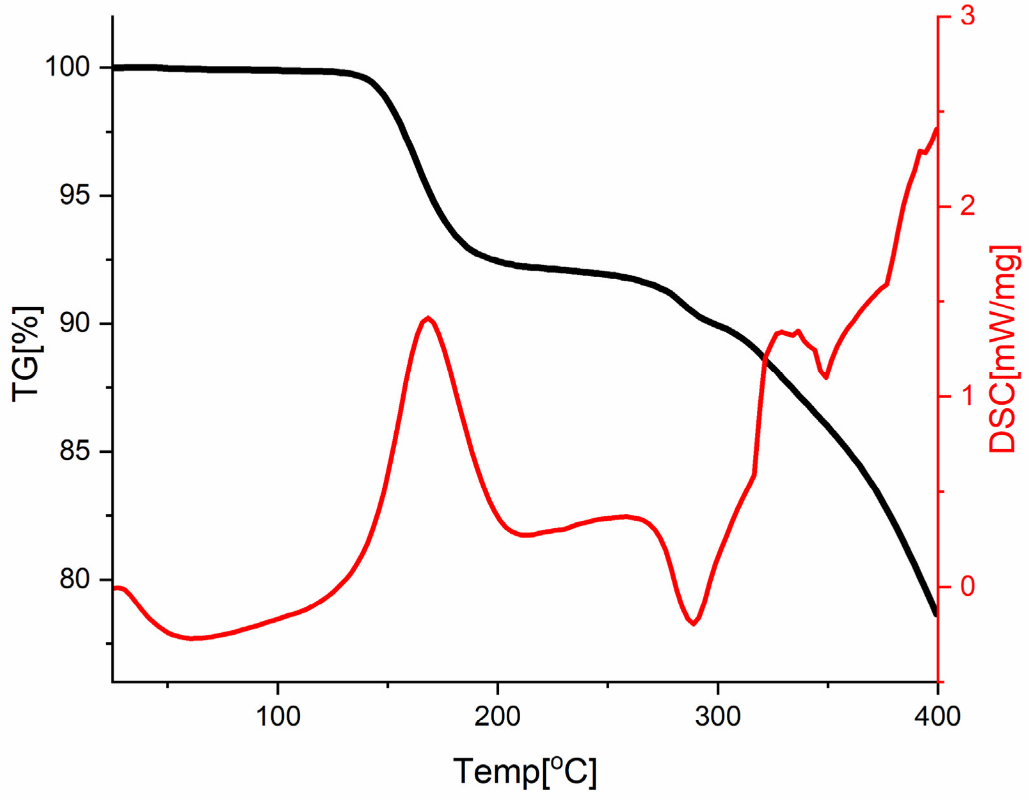

Figure S2. TG/DSC investigation for **K26dcpa**.

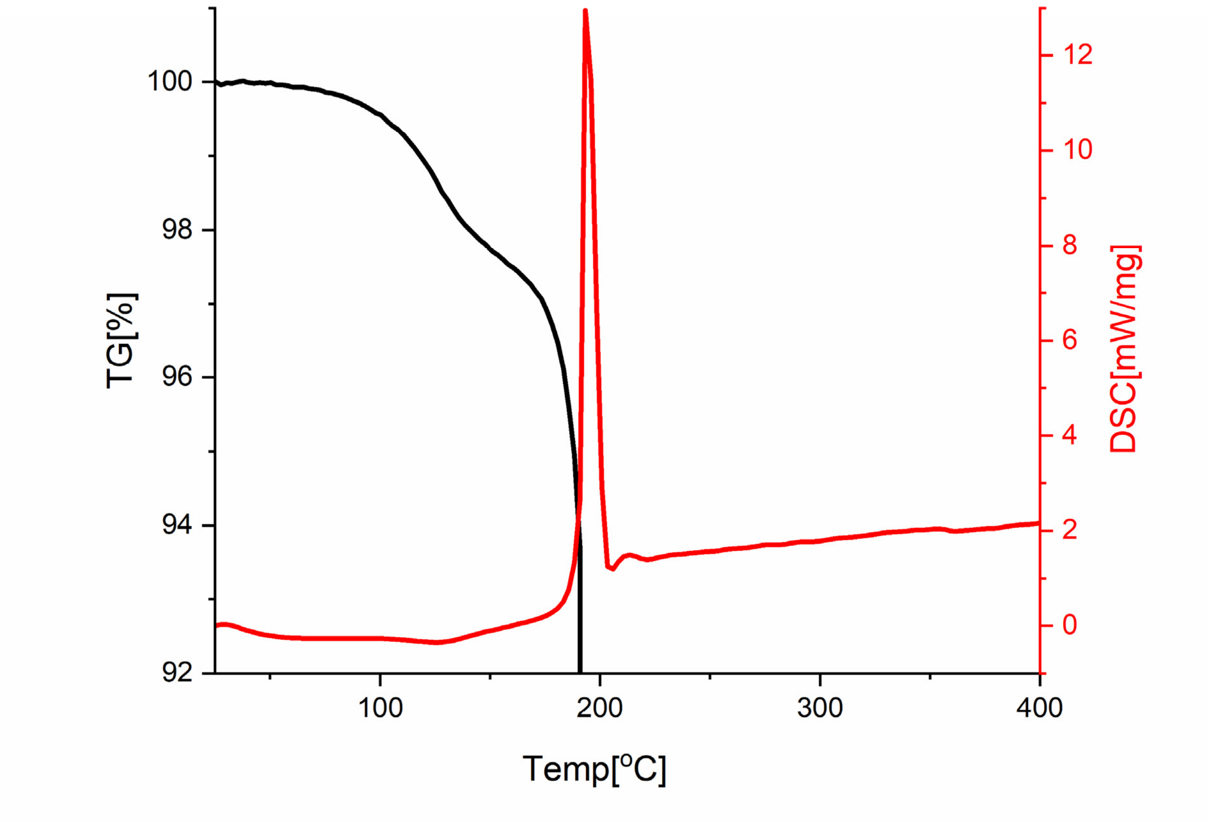

Figure S3. TG/DSC investigation for **K35dcpa**.

4. Catalysis for selected compounds comparing I, II, III run.

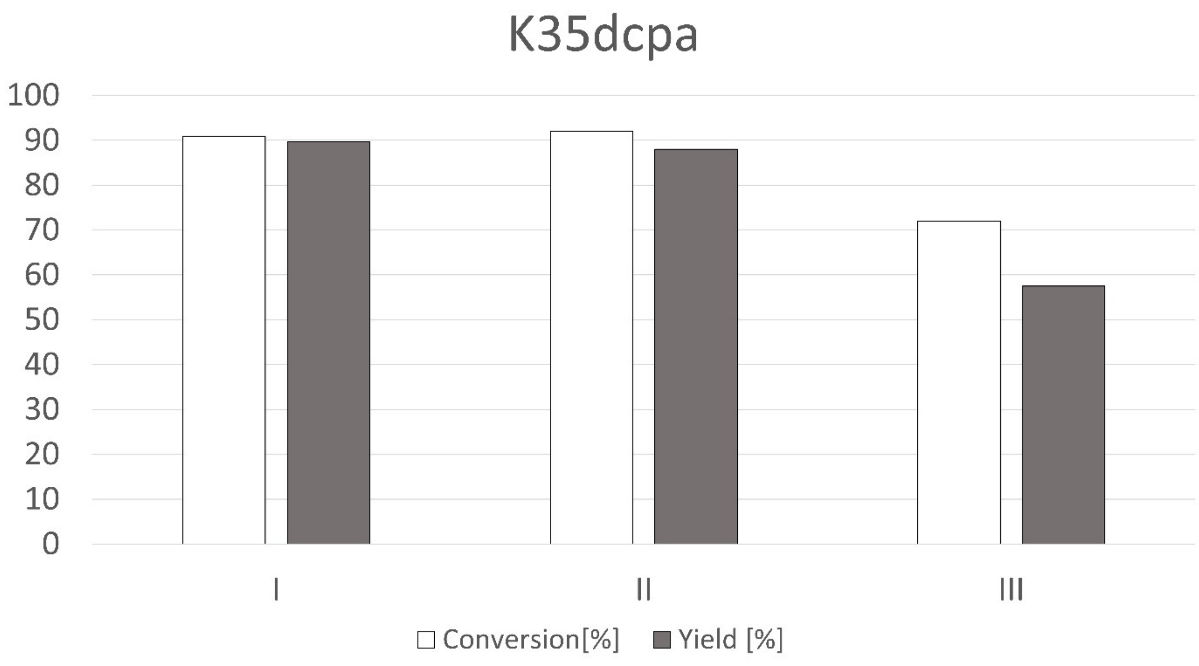

Figure S4. The BV oxidation of cyclohexanone with **K35dcpa** as catalyst in I, II, III run.

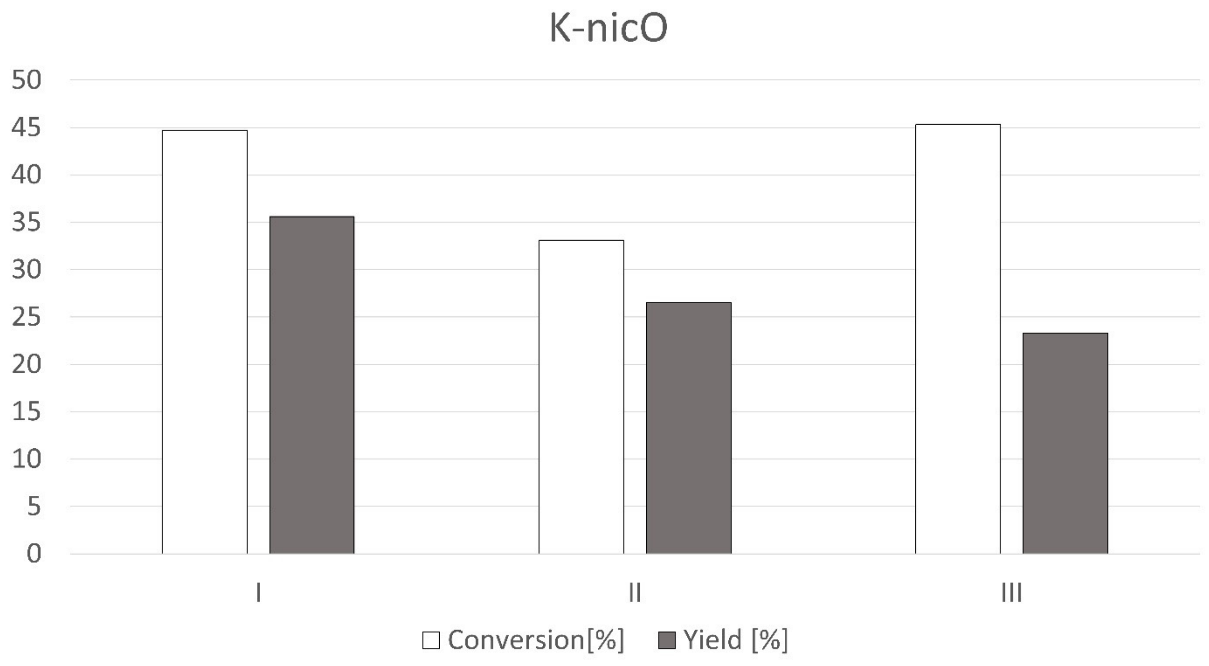

Figure S5. The BV oxidation of cyclohexanone with **K-nicO** as catalyst in I, II, III run.

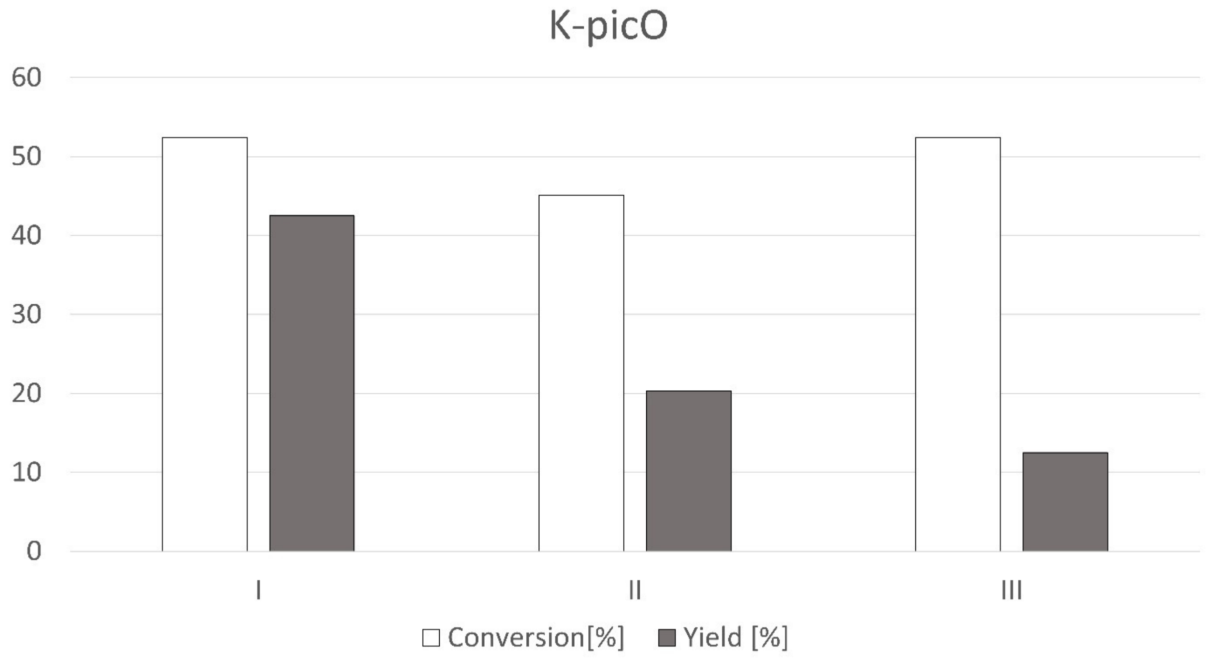

Figure S6. The BV oxidation of cyclohexanone with K-picO as catalyst in I, II, III run.

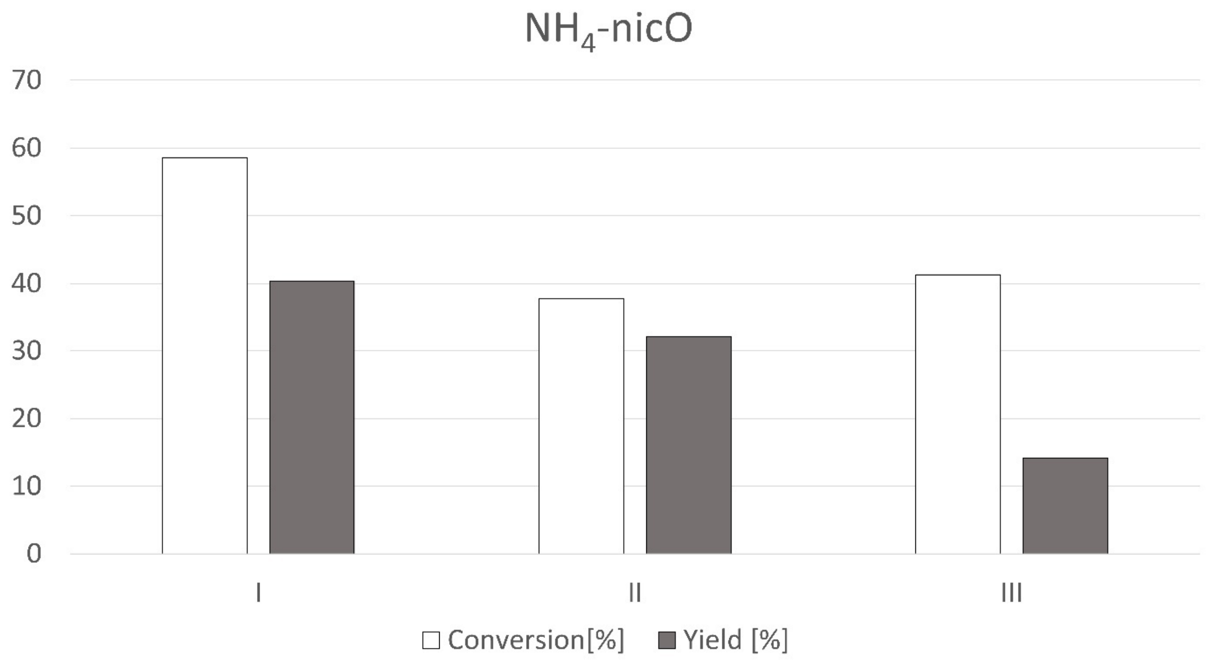

Figure S7. The BV oxidation of cyclohexanone with **NH_4-nicO** as catalyst in I, II, III run.

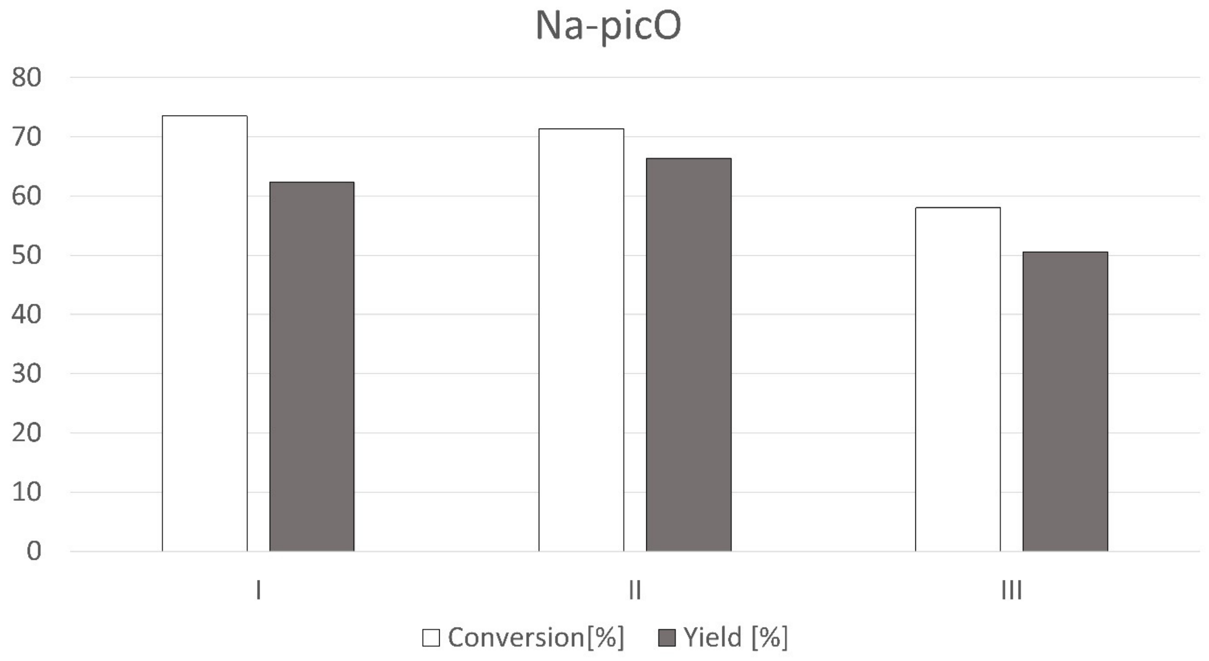

Figure S8. The BV oxidation of cyclohexanone with Na-picO as catalyst in I, II, III run.

5. X-ray diffraction, IR spectroscopy, an elemental analysis for selected compounds K-nicO, K-picO, NH_4-nicO, K35dcpa, K26dcpa and Na-picO before and after BV reaction.

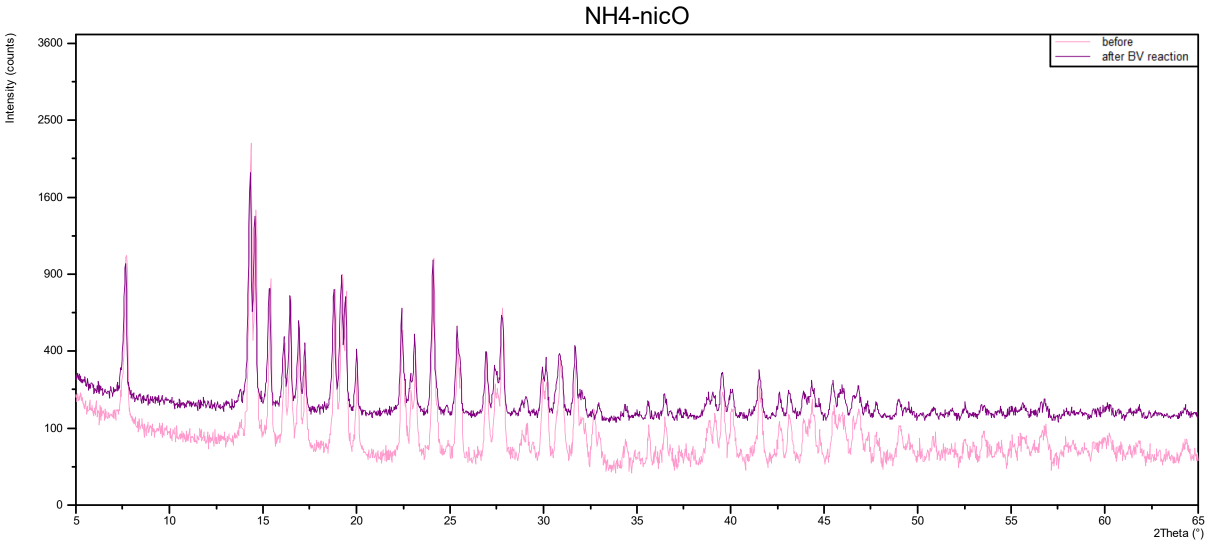

Figure S9. PXRD before and after BV reaction for **NH_4-nicO.**

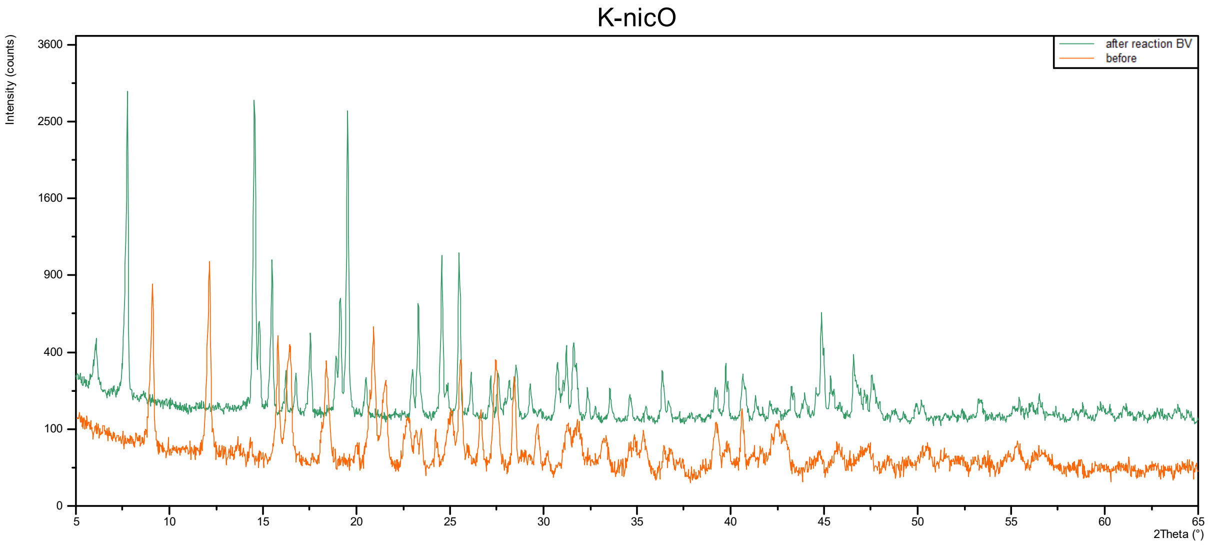

Figure S10. PXRD before and after BV reaction for K-nicO.

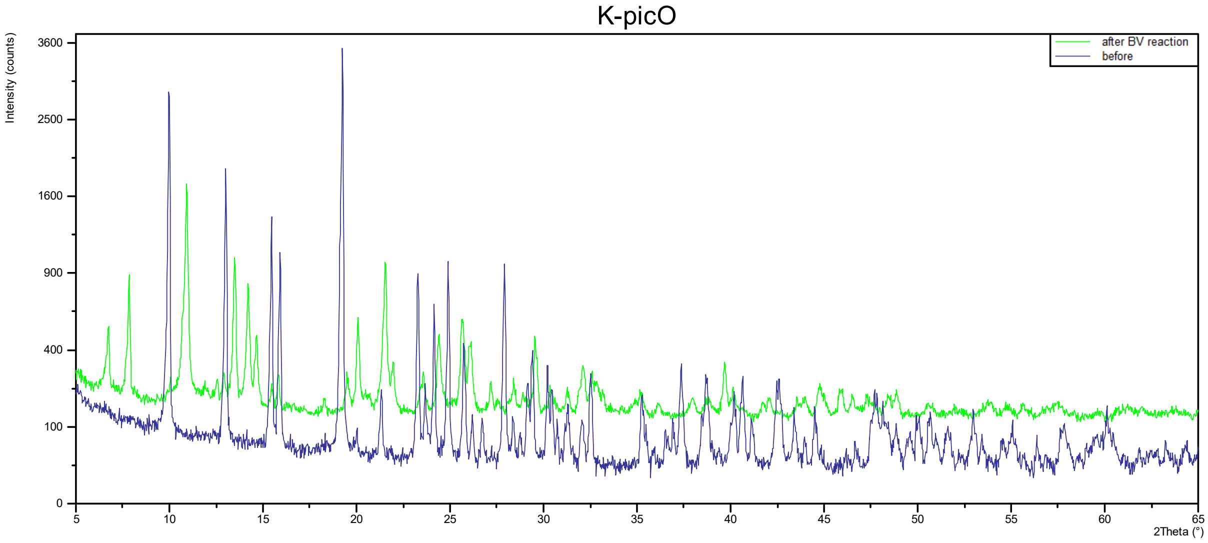

Figure S11. PXRD before and after BV reaction for K-picO.

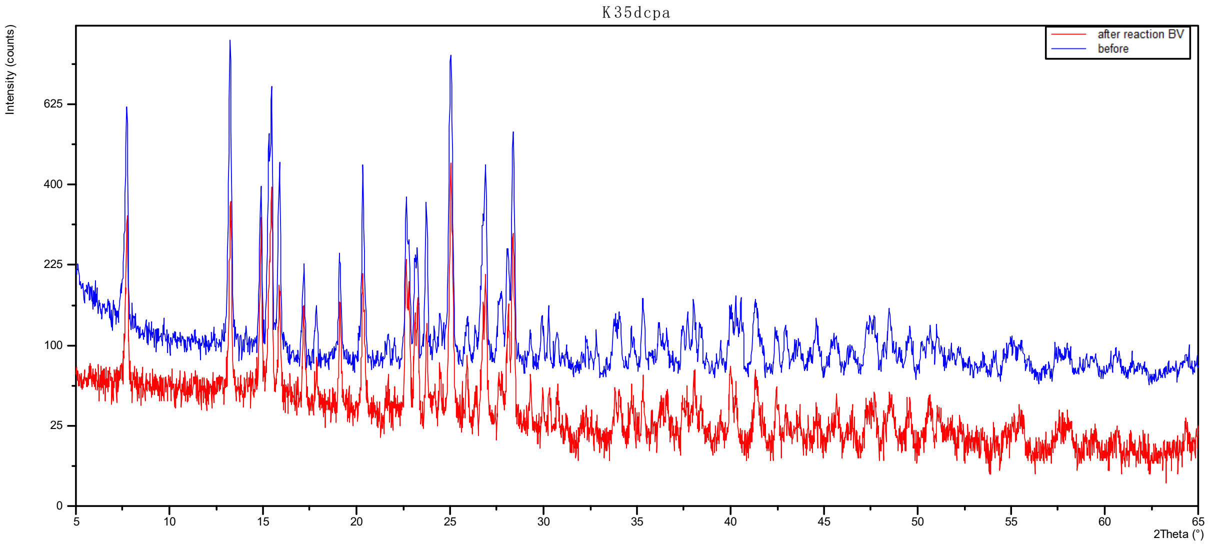

Figure S12. PXRD before and after BV reaction for **K35dcpa.**

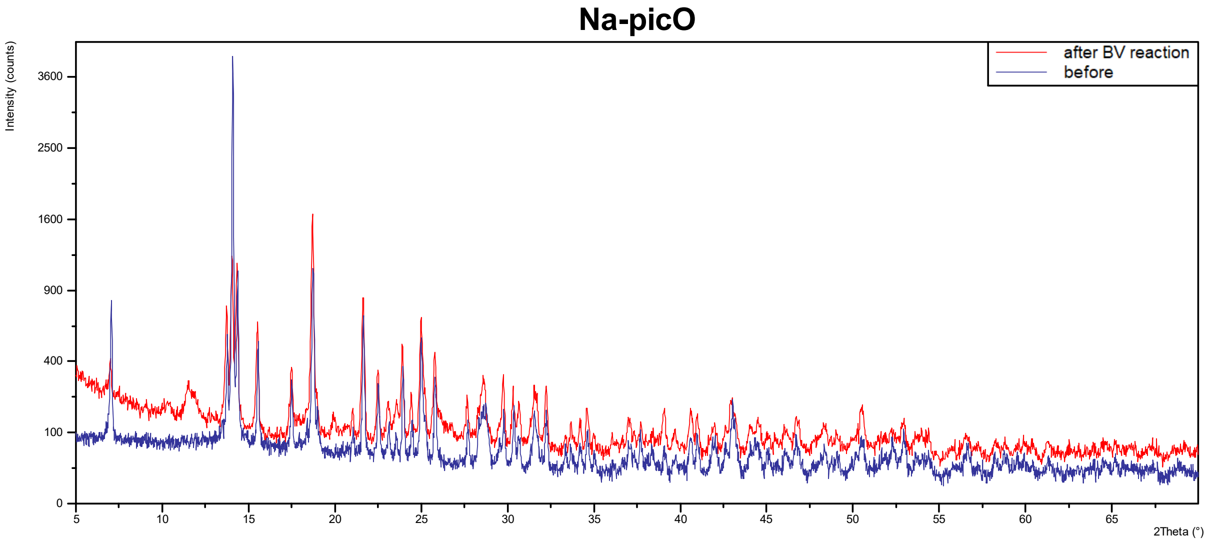

Figure S13. PXRD before and after BV reaction for Na-picO.

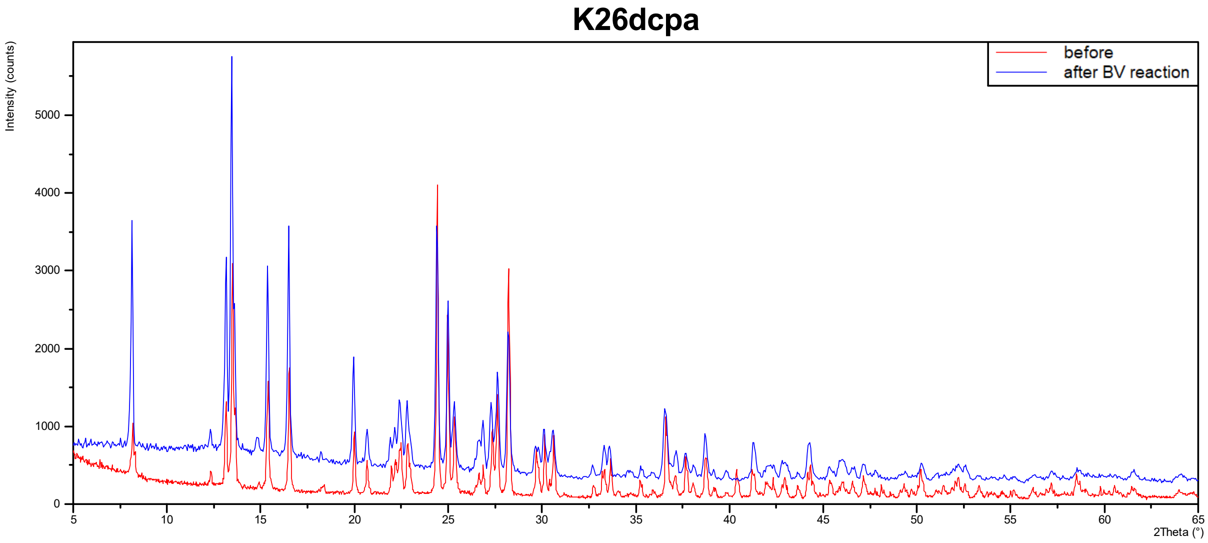

Figure S14. PXRD before and after BV reaction for **K26dcpa.**

Table S2. Result of elemental analysis for K-nicO and K-picO before and after BV reaction.

K-nicO		K-picO	
before	after (lost H_2O)	before	after
C 18.56 (calc. 19.42)	C 20.14 (calc. 20.41)	C 20.11 (calc. 20.41)	C 22.03
N 3.6 (calc. 3.77)	N 3.96 (calc. 3.97)	N 3.9 (calc. 3.97)	N 4.27
H 1.616 (calc. 1.63)	H 1.143 (calc. 1.14)	H 1.202 (calc. 1.14)	H 1.321

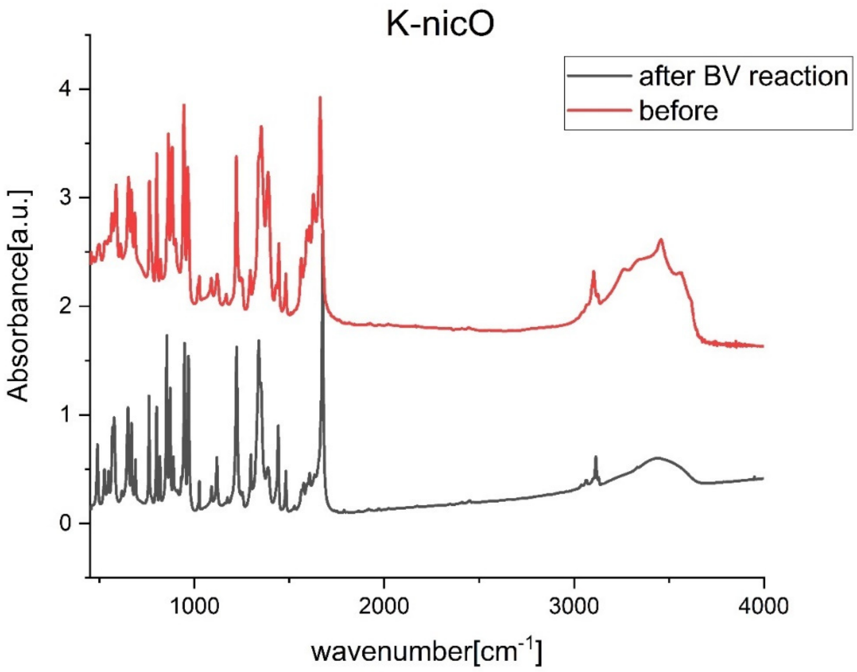

Figure S15. IR spectra of the compounds K-nicO before and after BV reaction.

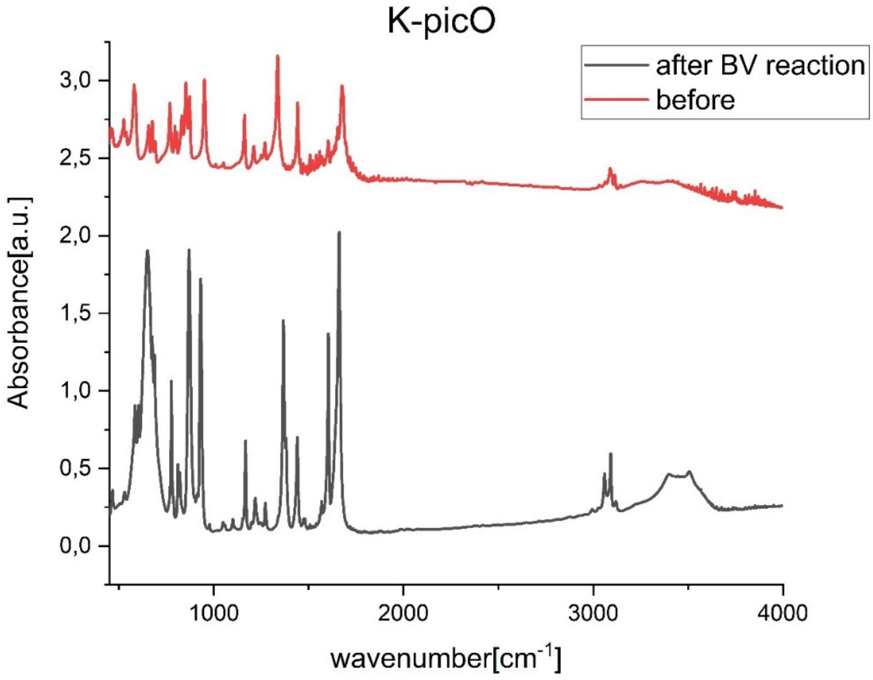

Figure S16. IR spectra of the compounds K-picO before and after BV reaction.

To conclude this part: Structure changes for *NH4-nicO*, **K35dcpa**, **K26dcpa** and *Na-picO* were not observed, changes in XRPD patterns were observed in the case of *K-nicO* and *K-picO*. However, chemical analysis and IR spectra (recorded for these 2 compounds) indicate only small changes in 'structure skeletons'. Probably the mild BV reaction conditions causes mostly only dehydration in the catalyst.
